# The utility of Zip4 codes in spatial epidemiological analysis

**DOI:** 10.1371/journal.pone.0285552

**Published:** 2023-05-31

**Authors:** Jayakrishnan Ajayakumar, Andrew Curtis, Jacqueline Curtis

**Affiliations:** GIS Health & Hazards Lab, Department of Population and Quantitative Health Sciences, School of Medicine, Case Western Reserve University, Cleveland, Ohio, United States of America; National University of Sciences and Technology NUST, PAKISTAN

## Abstract

There are many public health situations within the United States that require fine geographical scale data to effectively inform response and intervention strategies. However, a condition for accessing and analyzing such data, especially when multiple institutions are involved, is being able to preserve a degree of spatial privacy and confidentiality. Hospitals and state health departments, who are generally the custodians of these fine-scale health data, are sometimes understandably hesitant to collaborate with each other due to these concerns. This paper looks at the utility and pitfalls of using Zip4 codes, a data layer often included as it is believed to be “safe”, as a source for sharing fine-scale spatial health data that enables privacy preservation while maintaining a suitable precision for spatial analysis. While the Zip4 is widely supplied, researchers seldom utilize it. Nor is its spatial characteristics known by data guardians. To address this gap, we use the context of a near-real time spatial response to an emerging health threat to show how the Zip4 aggregation preserves an underlying spatial structure making it potentially suitable dataset for analysis. Our results suggest that based on the density of urbanization, Zip4 centroids are within 150 meters of the real location almost 99% of the time. Spatial analysis experiments performed on these Zip4 data suggest a far more insightful geographic output than if using more commonly used aggregation units such as street lines and census block groups. However, this improvement in analytical output comes at a spatial privy cost as Zip4 centroids have a higher potential of compromising spatial anonymity with 73% of addresses having a spatial k anonymity value less than 5 when compared to other aggregations. We conclude that while offers an exciting opportunity to share data between organizations, researchers and analysts need to be made aware of the potential for serious confidentiality violations.

## Introduction

Disease surveillance data or health outcome data, such as that collected by hospitals or made available to local health departments, can provide the opportunity for near-real time spatial analysis leading to on-the-ground improvements in healthcare [1]. These data might be used to target opioid overdose or gun injury clusters [2], as well as emerging infectious disease threats such as the recent Covid-19 outbreak [3]. Precise on-the-ground insights can support operational response including whether incorporating near-real time spatial syndromic surveillance [4], cross sectional cluster detection [5] or risk mapping [6]. For maximum intervention insight, for example identifying which congregate buildings are becoming “hot” with disease, it is vital to use granular data, such as a patient’s residential address, or the location of an overdose. This is not an unusual situation in that it mirrors typical emergency service response using 911 call for service data.

However, from a region served by multiple health systems, while such granular data exists within the Electronic Medical Record (EMR) or Emergency Department (ED) visit records, there is traditionally a barrier between institutions sharing their data because of spatial confidentiality and business concerns [7]. This concern is understandable as a plethora of works in spatial confidentiality research suggest that the use and sharing of personal geospatial information puts data contributors (research participants or even patients) at risk of being identified ([8–11]. This situation becomes even more untenable as other data attributes, or “quasi identifiers” are added to the geographic location [12]). And yet not finding an acceptable spatial analytical solution to data sharing at a granular geographic scale can inhibit an effective health response.

Geoscientists have tried to tackle spatial confidentiality challenges by developing spatial privacy protection policies or by manipulating the underlying geospatial data to preserve locational privacy [13]. While spatial privacy policies can help to alleviate privacy and confidentiality concerns, stringent policies such as no data sharing between research groups or health institutions can act as a major barrier for research collaborations, research reproducibility, and might also lead to repetitive data collection [14]. The type of constraints associated with spatial privacy policies lead to the development of geomasking/obfuscation techniques, which use mathematical transformation to transform geospatial data to protect locational privacy. While several variations exist such as donut masking [15], voronoi masking [16], and adaptive areal elimination [17] the complexities associated with implementing these types of advanced methodologies have led health institutions to primarily mask their data using spatial aggregation [18], which is the easiest to understand and implement.

While spatial aggregation to a postal delivery area (zip code) [19] census enumeration unit, or even a county can be easily achieved using software commonly available in a hospital system, the associated loss in the analytical power can have considerable operational consequences [7]. Simply put, the greater the aggregation, the less the risk of reengineering, but also a reduced insight in terms of detecting spatially specific risk through clustering [19, 20]. This tension is well known among geoscientists, and previous studies have tried to quantify the error involved, and the anonymity preserved while using aggregation based on commonly available spatial units such as County, Census enumeration unit, postal code, and census tracts. However, by comparison, little to no investigation has occurred regarding the use of one particular aggregation, the Zip4, which is an addition to the five-digit ZIP code of four additional numbers representing a geographic segment such as a city block, office building, an individual high-volume receiver of mail, or other distinct mail units. While the Zip4 geocode has been used as a source of spatial data in research [21–23], little is known about its operational strengths and weaknesses. This might be due to the lack of readily available polygon geometries that represent Zip4 codes. Indeed, while its geographic expression may vary, it is typically linear, not a commonly perceived form of spatial aggregation. This has led health institutions to extract out only postal code data (the five-digit zip code) from the Zip4 and use it for aggregation. In this paper, we assess the suitability of Zip4 codes as a source for transferring confidential locational data when viewed simultaneously through the lens of accuracy and confidentiality, through a series of comparative experiments with other commonly used sources of spatial aggregations and couch the results in terms of societal implications. The next section of this paper provides the necessary background regarding the utility of geospatial data in health research, the spatial confidentiality risk associated with health data, and the development of techniques to preserve spatial confidentiality while preserving the usability of the data. After that, we provide information about data and experimental setup followed by the results and discussions. The final section of the paper will discuss the conclusions from this study.

## Background

While the power of analysing geographic health data is widely understood by spatial scientists [24–26], that same appreciation has only relatively recently expanded (and continues to grow) within clinical and public health organizations. For example, a hospital system might develop spatially guided precision medicine strategies [27] using their own internal data. Typical tasks would include mapping patient locations [28] or finding distances between patients and nearest facilities or services [24]. At a more elevated level, recent developments in spatial data storage systems such as spatial databases [29], and novel spatial analysis techniques such as spatial and spatio-temporal clustering [30], have enabled researchers and health practitioners to utilize health data for developing real-time spatial support systems [1]. These spatial support systems can be particularly useful in balancing surge across multiple hospitals based on where events happen, such as gunshots in a building complex. From an infectious disease perspective, monitoring the spread of cases across space and time can help inform clinicians and health authorities about potential outbreaks and mobilize preventive strategies [31–33] or predict where upcoming surge could stress resources. For example, in northeast Ohio a spatial syndromic surveillance system [4, 34, 35] was used to identify emerging Covid-19 disease clusters at a granularity that includes care homes and congregate living buildings. The results were used to guide hospital intercept teams to limit further disease spread both within and between densely packed living areas. Even though there has been an appreciation among health organization regarding the value of this type of fine-scale spatial insight, the issue of spatial privacy and confidentiality has always been an impedance for data sharing and collaborations between health organizations.

The spatial confidentiality risk associated with using geographically granular data or how inappropriate mapping might lead to an unacceptable re-engineering of location are well known [36–39]. Suggested solutions have included the use of anonymity, spatial privacy policies (such as HIPPA), and obfuscation/geomasking [40]. Of these, geomasking is the most commonly applied by health organizations, and the most likely to be implemented in the high-pressure, short time frame of a regional response to an emerging disease threat. More specifically, Geomasking can be categorized into affine [7, 41], random perturbation [15] or spatial aggregation [17]. Affine geomasks utilize geometrical translations, rotation, or a combination of both for relocating non-aggregated point data. Random perturbation displaces the original point either deterministically or stochastically. Implementations include grid masking [42, 43], “flipping methodology” [44], donut masking [15, 45], Vornoi masking [46], verified neighbor masking [47], and adaptive areal elimination masking [17]. Due to the complexities associated with implementing advanced geomasking methods, health organizations have always adopted geomasking based on spatial aggregation. While easier to implement, geomasking based on spatial aggregation leads to loss in analytical accuracy especially at coarse scales [41, 48, 49].

Research on the effects geographic masking has on different spatial-analytic methods is quintessential to determine whether the masking or aggregation strategy that is being applied strikes an appropriate balance between the protection of confidentiality and the ability to derive relevant spatial relationships [13]. Kwan and associates [41] in their study to examine the effects of two different masking techniques: random direction with a fixed radius and random placement within a circle, had shown that there was a consistent tradeoff between the amount of spatial manipulation and the accuracy of the analytical results. Another study using artificial clusters generated from a SaTScan of point locations masked using bimodal Gaussian displacement suggests that there was a gradual decrease in cluster detection sensitivity and specificity with an increase in the average displacement distance [50]. Similarly, another study on household travel surveys where donut masking was used as the geomasking method revealed that there was a gradual reduction in analytic accuracy with increasing displacement between points [51]. Lu et al. [52] in their work on measuring the impact of aggregation based masking on spatial analytic methods such as Nearest Neighbor Index and Moran’s I found that there were only minor effects associated with geographic masking for displacements of up to 250 m. Recently, Wang et al. [53], did an extensive comparative analysis between eight different geographic masking methods including aggregation to tease out the efficiency of geomasking in privacy protection while maintaining sufficient analytical accuracy. Their results suggests that geomasking methods such as point aggregation introduces error as the masking radius increases. While the research on geomasking using spatial aggregations have focused on quantifying the error introduced with readily available aggregation units such as zip code or Census tracts, there is a dearth of research in using other aggregation units such as Zip4 codes or street segments. To address this gap, we assess the usability of Zip4 codes as a spatial unit for aggregation based geomasking through a set of comparative experiments.

## Data

### Zip4 address data

#### Acquiring Zip4 address data

Currently there are no official data sources available for obtaining Zip4 address data. Even though the United States Postal Service (USPS) provides access to a reference file that can be used to assign a Zip4 to a physical address [54], the process of obtaining the file and further parsing the Zip4 addresses is not trivial. Apart from the USPS, many private vendors such as Melissa (https://www.melissa.com/v2/lookups/zip4/zip4/) provide Zip4 API lookups and Zip4 address data extracts, often at a considerable cost. Large address sources such as OpenAddresses (https://openaddresses.io/) provide residential addresses for places, which can also be used for mining Zip4 records during the geocoding process. For this study, we collected a large data extract of 1.6 million Zip4 codes within the state of Ohio from a private vendor without any cost. The data extract was available as a large tab separated text file, which was then converted into a data table after preprocessing. Each row of the data table has a unique Zip4 code as well as additional attributes such as the upper, and lower level street address associated with the Zip4 code. Table 1 shows the structure of a Zip4 record along with its various attributes. The lower address for the first row is “4459 E Main St Cleveland, 44106” and the higher address is “4477 E Main St Cleveland, 44106”, while the low and high address for the second row is 7453 Sadie Rd N Akron, 43001. Generally, Zip4 codes that have the same lower and higher address tend to be congregate living facilities such as apartments, nursing homes, care homes etc.

**Table 1:** Zip4 record with attributes.

Zip	Plus4	City	PreDir	Street	Suffix	PostDir	Address Low	Address High
44106	3138	Cleveland	E	Main	St		4459	4477
43001	0015	Akron		Sadie	Rd	N	7453	7453

#### Geocoding Zip4 data

While some Zip4 data may be mapped as a point if enough addresses are found in a single building, more typically the output are polylines that require additional manipulation if they are to be used for spatial analysis. To do this, the lower and higher addresses associated with the Zip4 code were geocoded using ArcGIS which includes as output “Point Address” (house and building locations), “Street Address”, “Street Name” (missing house number), “Locality” (place-name representing a populated place), “Postal” (five digit Zip code), and “Admin” (state name). Here we only use the Zip4 records that can either be geocoded to the “Point Address” or “Street Address” to maximize precision. The lower and higher addresses are separately geocoded and the resulting shapefiles are merged together using the Zip4 code as the common key. In order to efficiently retrieve and perform a geometrical operation on the addresses, a spatial database (PostgreSQL, with PostGIS extension) was developed. Finally, each Zip4 code along with the line segment joining the coordinates of the lower and higher address was stored as a geometry object to the spatial database. A total of 1,389,482 Zip4 records were geocoded at either ‘Street Address’ or ‘Point Address’. In this paper, to mirror the geographic area analyzed by the authors as part of a multi-hospital response to Covid-19 response, only Zip4 records for Cuyahoga County and Portage County were included. These were also selected because Cuyahoga County is more urban in nature, with its major urban center being Cleveland, while Portage County has small less dense settlements and an overall “rural” nature. Using the described approach there were 137,732 Zip4 records geocoded for Cuyahoga County and 16,639 records for Portage County.

### Voter data

In order to understand the potential of using Zip4 centroids for analytical purposes, Ohio voter data (from now on OV) was utilized as a proxy for typical patient data. The OV dataset is freely available to the public through the Ohiosos (Ohio Secretary of State) website (https://www.ohiosos.gov/). This dataset contains 8,070,402 records with details such as the political affiliation and residential address of voters. ArcGIS was used to geocode 7,932,791 (98%) of these addresses to either the Point or Street level. All the geocoded addresses were added to the spatial database to enable faster spatial queries and other geometric manipulations. Similar to the Zip4 address data, only voters address data that were within Cuyahoga County (n = 773,334) and Portage County (n = 83,778) were further used for the analysis.

### Data for comparative spatial analysis

In order to compare different spatial aggregations, the OV dataset was spatially aggregated by street segment (from now on SS), Census Block Group (from now on CBG), and Zip code polygons. OpenStreetMap (OSM) [55], a collaborative project to create free editable maps, provides various free geodata including street segment data. To download street segment data for Cuyahoga County and Portage County we utilized OSMnx [56], which is an open-source tool built in Python for querying OSM API’s. The street segment data extracted using OSMnx were added to the spatial database as a line segment object and its centroid was used in the analysis. The OV dataset was aggregated to CBG and Zip spatial units through a point-in-polygon operation. For SS aggregation, each voter record was assigned to its nearest SS centroid. For Zip4 aggregation, the reverse geocoding option available in ArcGIS, which converts a coordinate to a physical address, and generates the Zip4 code associated with that location, was utilized. Each of the records in the OV dataset were assigned a Zip4 code through this reverse geocoding process and the associated geographical coordinates (Zip4 centroid) were retrieved from the spatial database and assigned to each of the records. Finally, a spatial table was generated, with each row containing a voter’s original address in coordinates (real), Zip4 code, Zip4 centroid, SS centroid, CBG centroid, and Zip centroid (Table 2).

**Table 2:** An example of voter data with real, Zip4, Street Segment, Census Block Group, and Zip centroid details.

Vid	Lon, Lat	Z4Lon, Z4Lat	SSLon, SSLat	CBGLon, CBGLat	ZLon, ZLat	Zip4 Code
1	-81.345,42.564	-81.344,42.563	-81.315,42.524	-81.245,42.564	-81.137,42.434	44106–1234
2	-81.632,42.233	-81.631,42.255	-81.621,42.225	-81.344,42.123	-81.223,42.112	44118–1134

### Data for clustering analysis

In order to replicate the type of analysis performed during the Covid-19 pandemic response, a synthetic dataset was created from the Cuyahoga County OV records. Case and control data required for the spatial clustering methods were sampled from the dataset based on the Covid-19 test result distribution for Cuyahoga County. To achieve this, the boundary polygon for Cuyahoga County was tessellated into 5km grids and the total number of negative and positive Covid-19 test results that fell into each grid was calculated using a point-in-polygon operation. For the spatial clustering analysis, 1,000 positive cases and 3,969 negative cases were sampled from the dataset using the test result distribution.

## Experimental setup

### Zip4 length distribution

**Fig 1:**
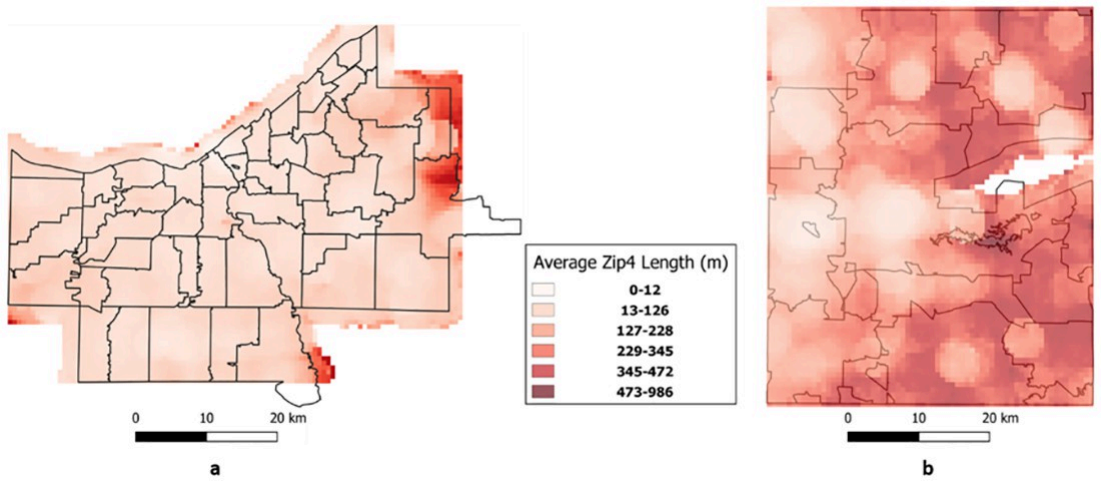
Spatial distribution of length of Zip4 segments for (a) Cuyahoga County, and (b) Portage County. Zip Code boundaries are also displayed.

Typically, Zip4 codes are represented as polylines with an upper and lower address representing the two ends. To understand the distribution of these lengths, a histogram-based approach was followed. The length of each polyline was calculated using the in-built length function in PostGIS and then the results were aggregated in intervals of 100 meters. Further, the percentage of records that fall into each bins were calculated. While a histogram provides a general trend of the length distribution for Zip4 codes, it does not provide any spatial insights. To understand the spatial distribution of lengths of Zip4 codes a grid-based approach was followed (Fig 1). The cell size for the grid was 500 meters and the bandwidth (radius) was 2000 meters. Each cell was assigned a value based on the average length of Zip4 polylines that fell within the 2000m radius. Both these experiments were conducted using the Zip4 data from Cuyahoga and Portage counties.

### Distance to real location

In order to establish how much potential analytical error occurs when using the Zip4 centroids, the distance between the centroid and the address location was calculated. These calculated distances were summarized as a histogram with a bin size of 50 meters for Cuyahoga and Portage counties. Further, the total counts in each bin were normalized using the total number of records.

### Spatial analysis

To test the impact of using Zip4 centroids as compared to “real” data, a series of experiments were conducted on the OV dataset for Cuyahoga County. As there were a large number of addresses for Cuyahoga County (n = 773,334), smaller subsets of randomly permuted address points were used for the experiments; 100 datasets each containing 10,000 randomly selected address points were generated and used for further analysis.

#### Average nearest neighbor analysis (ANN)

The average nearest neighbor analysis helps to determine whether a point pattern is clustered or not [57]. A point pattern is deemed clustered if the average of the nearest neighbor distances is less when compared to the average of a hypothetical random distribution. For this experiment, ANN was run on each of the 100 datasets for real, Zip4, SS, CBG, and Zip centroids.

#### Ripley’s K

While ANN analysis identifies clustering at a global level, Ripley’s K function [58] helps to determine whether clustering occurs at various local distance bands. Ripley’s K function was applied to the datasets in each category and the observed and expected counts at various distance bands were recorded.

#### Kernel Density Estimate

**Fig 2:**
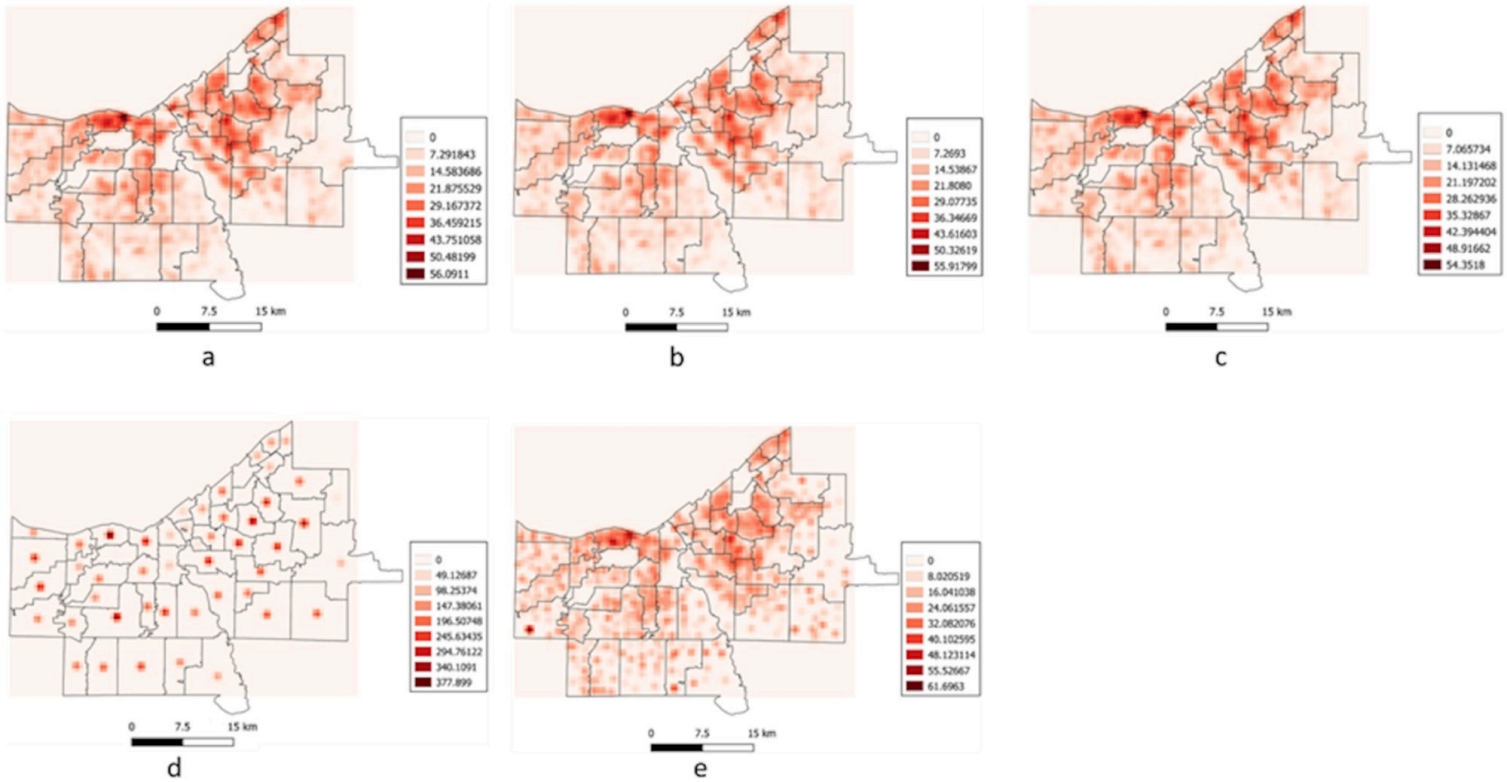
Kernel Density Estimate (KDE) surfaces generated for (a) Real, (b) Zip4, (c) Street Segment, (d) Zip, and (e) Census Block Group.

Kernel Density Estimation (KDE) [59] calculates the density of features around a point of interest. The area of interest (in this case Cuyahoga County) is covered with a grid surface (here 500 meters) and the density across each grid cell is calculated by identifying all the points that fall within a bandwidth (here 1000 meters). KDE was run on the 100 datasets for the five categories (Fig 2), and the raster surface generated was compared to the raster for original dataset by calculating a cell-to-cell correlation coefficient.

#### Cluster detection

An important part of syndromic surveillance is spatial cluster detection [4, 32, 60]. During the Covid-19 pandemic identifying emergent clusters was a key approach used by hospital systems in Ohio to respond to outbreaks [4]. The type of spatial clustering developed for Ohio is extremely sensitive to geocoded accuracy and precision. To understand how Zip4 centroid aggregation affects spatial clustering and detection, two different spatial clustering methods were used; SaTScan [61] and GeoMEDD [4]. SaTScan is a popular spatial clustering methodology used by epidemiologists and health geographers around the world to detect spatial clusters of infectious and chronic disease [62], as well as disease vectors and risk factors [63]. For cluster detection, SaTScan uses the spatial scan technique, which creates a theoretically limitless number of circles in a geographic area [61]. The circles vary in size and each circle is evaluated as a possible cluster by being compared with the area outside the circle. The circle with the highest maximum likelihood of being a cluster is assigned a *P* value by using a Monte Carlo Simulation. GeoMEDD [4] is a recently developed spatial clustering methodology based on DBSCAN [64] that groups proximate points based on both spatial and temporal distance. GeoMEDD clusters used to identify emerging threats are typically classified into sentinel (a minimum of two members within a distance of 100m), micro (a minimum of five members within a distance of 500m), and neighborhood clusters (a minimum of 10 members within a distance of 1000m) [4] for 3 and 7 day lookback periods. Unlike SaTScan, which generates only circular clusters, GeoMEDD generates clusters of any shape based on the location of cluster members being connected by a convex hull.

**Fig 3:**
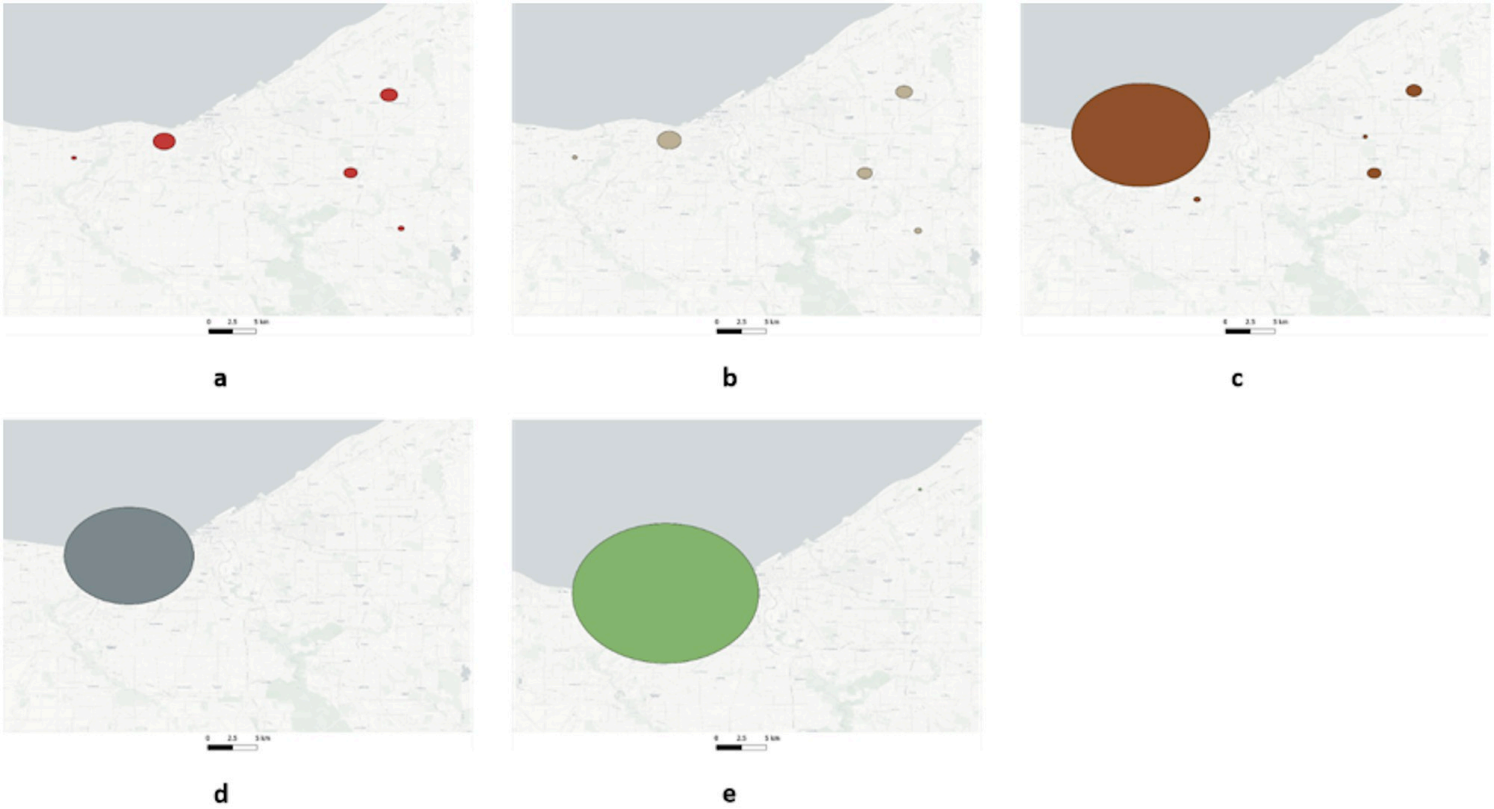
SaTScan clusters for (a) Real, (b) Zip4, (c) Street Segment, (d) Census Block Group, and (e) Zip.

**Fig 4:**
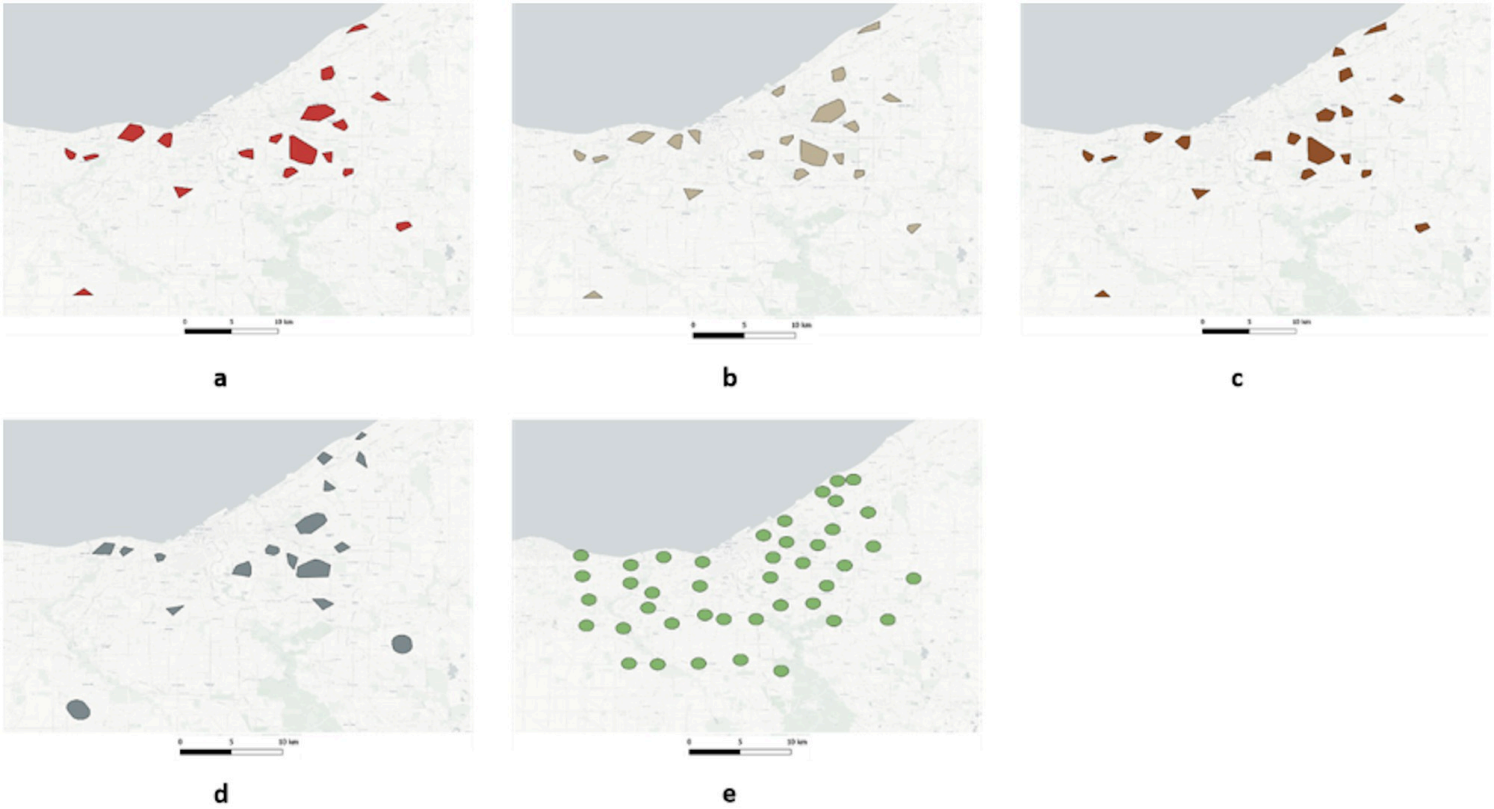
GeoMEDD clusters for (a) Real, (b) Zip4, (c) Street Segment, (d) Census Block Group, and (e) Zip.

To understand how Zip4 based aggregation changes spatial cluster detection, a comparative analysis was conducted. Five different locational datasets including the geographical coordinates of the real dataset, and the centroids for Zip4, SS, CBG, and Zip were created. SaTScan was configured to detect purely spatial clusters based on the Bernoulli model (case-control). For the same case and control data, SaTScan runs were made for the five different locational datasets (Fig 3). Each run generated cluster outputs as ESRI shapefiles (Polygon), Keyhole Markup Language (KML) files, and a summary text file. For GeoMEDD, the neighbourhood clustering (10 member and 1000 meter) was run for the five locational datasets (Fig 4). The cluster member identifiers were stored as JavaScript Object Notation (JSON) files while the cluster geometries (Polygons) were stored as ESRI shapefiles.

To quantify the similarity between the datasets, the individual cluster member details were used. The cluster members for all the categories were compared against the real category using the Jaccard similarity coefficient. Jaccard similarity coefficient for two sets is defined as the ratio between the size of the intersection and the size of the union between the sets. The Jaccard similarity coefficient of the real cluster to the corresponding aggregated cluster was calculated and its average was assigned as the similarity score. A similarity score of one indicates perfect similarity while a value of zero indicates complete dissimilarity. Apart from this similarity score, the total number of real clusters that were never detected as well as the total number of spurious clusters generated as an artefact of the aggregation was also noted. The missing and additional clusters were identified by applying a spatial intersection test between each of the real clusters and the aggregated clusters.

### Privacy

#### Re-engineering risk with Zip4 aggregation

To address the re-engineering risk of using a Zip4 based aggregation, the percentage of points falling within a distance of 5 meters (as a minimum tolerance) to the assigned Zip4 centroid was calculated. For a comparative analysis, the same procedure was repeated for SS, CGB, and Zip categories.

#### Spatial K-anonymity

**Fig 5:**
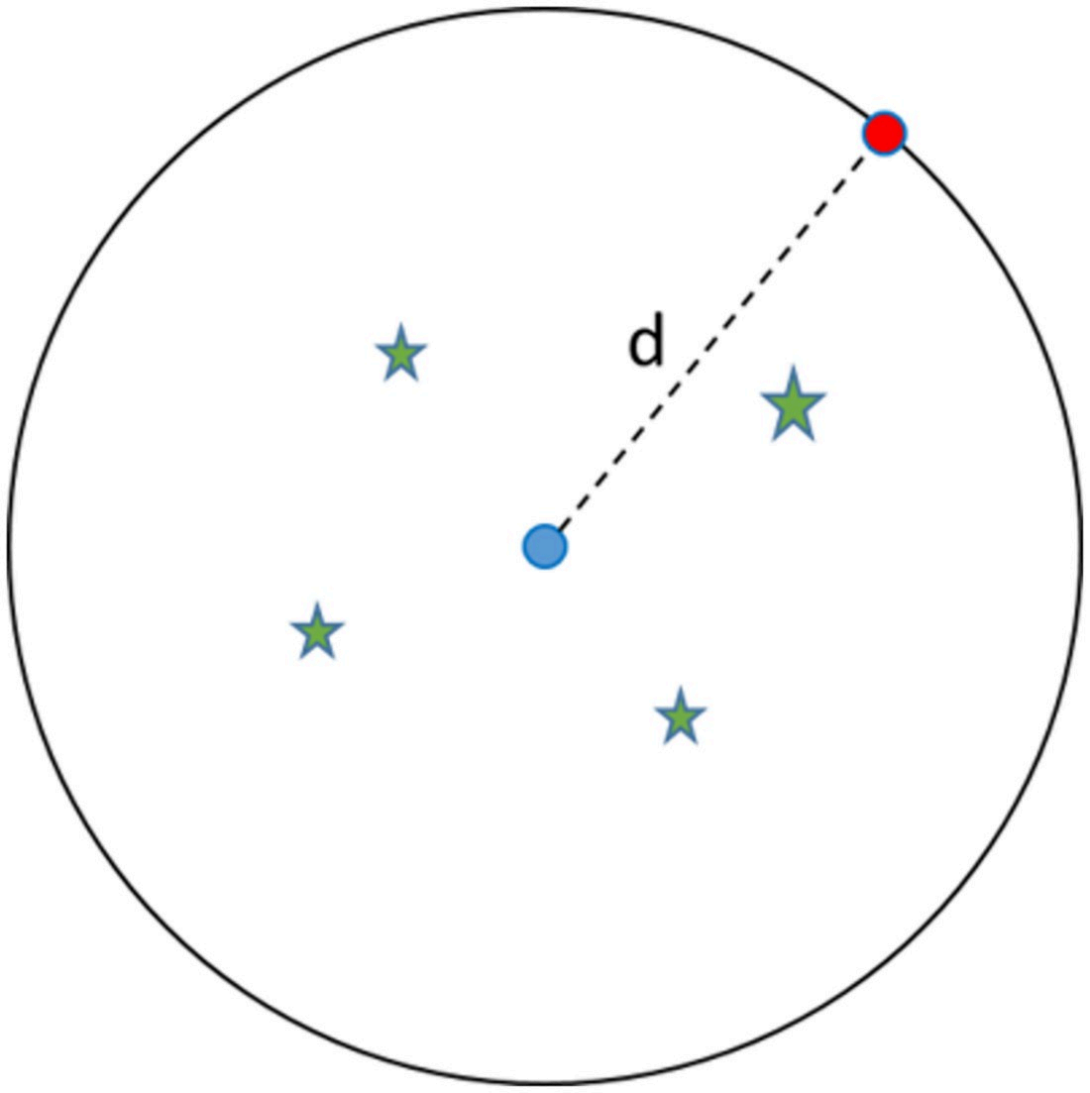
Spatial K-anonymity calculations. The red point is the original location and the blue point is the geomasked location. The green stars are other potential locations.

Spatial K-Anonymity [65] is a widely adopted method to evaluate the degree of geoprivacy achieved with any type of geomasking technique. It is a variant of k-anonymity [66] which quantitatively assess the probability of identifying an individual record from a group of other records. Spatial k-anonymity estimates the probability of identifying an individual location from a set of locations after geomasking. The k value in k-anonymity is determined by calculating the total number of other (k-1) addresses that fall within the region (circular) between the real and the new anonymized location [14]. An example of calculating k-anonymity for a single location is shown in Fig 5. Higher k value indicates increased spatial privacy preservation as the probability of re-identification (given as 1/k) decreases with an increase in k.

In order to calculate k-anonymity when using a Zip4 centroid, we used the Cuyahoga County address data obtained from the Cuyahoga County Open Data portal (https://data-cuyahoga.opendata.arcgis.com/). The dataset contains 490,453 address locations in Cuyahoga County with their respective geographical coordinates. The k-anonymity value for each location is calculated as per the methodology described in Fig 5 and the k-anonymity value for the dataset is calculated by taking the average k value across the entire dataset. For a comparative analysis, k-anonymization was also implemented on the SS and CBG datasets.

## Results

### Zip4 length distribution

The Zip4 length distribution experiment revealed that Portage County had 67% of its Zip4 addresses with a length less than 200 meters, and 91% were within 400 meters (Table 3). By contrast, 91% of the Zip4 addresses in Cuyahoga County had line segments of less than 200 meters and 97% were within 400 meters (Table 3).

**Table 3:** Zip4 length distribution (in meters) for Cuyahoga and Portage counties.

County	0–100	101–200	201–300	301–400	401–500	501–600
Portage	46%	21%	16%	8%	2%	1%
Cuyahoga	76%	15%	5%	1%	0.3%	0.1%

From the grid surface, which shows the spatial distribution of Zip4 lengths (Fig 1), it could be gleaned that Portage County generally had longer Zip4 line segments, and that there was also a west-to-east gradient with the eastern side being more typically rural. Cuyahoga County appears more homogenous though there is a less dense band on the eastern edge.

### Distance to real location

The histograms for the distance between Zip4 centroid and real location revealed that for Cuyahoga County 81% of the voter address locations were within 50 meters of the associated Zip4 centroid and 99% of the addresses were within 150 meters (Table 4). In Portage County 67% of the voter addresses fell within 50 meters of their respective Zip4 centroid and 91% of the addresses were within 150 meters.

**Table 4:** Distance distribution (in meters) between real location and Zip4 centroid for Cuyahoga and Portage counties.

County	0–50	51–100	101–150	151–200	201–250	251–300	301–350	351–400
Cuyahoga	81%	16%	2%	0.3%	0.1%	0.06%	0.04%	0.02%
Portage	67%	24%	5%	1%	0.6%	0.3%	0.2%	0.1%

### Spatial analysis

#### Average nearest neighbour analysis (ANN)

The results from Average Nearest Neighbour Analysis shows that the real locations exhibits clustering and has an average nearest neighbour value (NNR) of 0.55 (Table 5). Zip4 (0.52) and SS (0.48) have similar NNR values while CBG (0.005) and Zip (0.001) differ considerably when compared to the real locations. The Pearson Correlation Coefficient between the NNR values for real and other categories reveal that the Zip4 NNR values are closely correlated to real category NNR values (0.91), followed by SS (0.76), CBG (0.1), and Zip (0.1) (Table 5).

**Table 5:** Average Nearest Neighbour analysis results for real and centroid datasets. The NNR is calculated as the average for 100 datasets.

Category	NNR (Mean 100 datasets)	Pearson Correlation Coefficient To Real
Real	0.55	1
Zip4	0.52	0.91
Zip	0.001	0.1
SS	0.48	0.76
CBG	0.005	0.1

#### Ripley’s K

The results from the Ripley’s K analysis (Table 6) suggest that the observed values for the real category at various distance bands are similar to the observed values of Zip4 and SS and dissimilar to Zip and CBG categories.

**Table 6:** Ripley’s K results using the real and centroid datasets.

Expected K	Observed (Real)	Observed (Zip4)	Observed (SS)	Observed (CBG)	Observed (Zip)
1000	1589	1588	1588	1470	3281
1200	1864	1863	1863	1733	3281
1400	2133	2132	2131	1982	3281
1600	2394	2394	2392	2226	3281
1800	2650	2649	2647	2484	3281
2000	2901	2900	2898	2725	3281
2200	3148	3147	3145	2961	3305
2400	3392	3391	3389	3196	3335
2600	3634	3633	3631	3433	3341
2800	3874	3873	3871	3658	3493
3000	4111	4110	4108	3883	3524

#### Kernel density estimate

The results of the KDE runs are shown in Fig 2. The results of cell-to-cell correlation coefficient (Table 7) reveals that Zip4 (0.99) and SS (0.99) category had an identical distribution to the real category, while CBG had a highly similar distribution but Zip was highly dissimilar when compared to the real category.

**Table 7:** KDE raster cell comparison between real and centroid datasets.

Category	Correlation Coefficient
CBG	0.88
SS	0.99
Zip	0.19
Zip4	0.99

#### Cluster detection

The SaTScan results using the real dataset (Fig 3a) revealed that five clusters were generated. The clusters generated with the Zip4 dataset (Fig 3b), produces the closest pattern of clusters generated using the real dataset, followed by clusters generated from the SS dataset (Fig 3c). The cluster results from the CBG (Fig 3d) and Zip (Fig 3e) clearly reveal that, an increase in aggregation led to an increase in missing clusters. The GeoMEDD clustering methodology generated 18 clusters across Cuyahoga County, and a visual examination of the output maps reveal that Zip4 clusters (Fig 4b) are highly similar to the real dataset (Fig 4a) followed by SS (Fig 4c), CBG (Fig 4d), and Zip (Fig 4e) clusters.

The similarity scores (Table 8) for the SaTScan clusters reveal that the Zip4 clusters are highly similar (0.89) to the real clusters in terms of the cluster members followed by SS (0.29), CBG (0.01) and Zip clusters (0.0). Apart from being highly similar to the real clusters, there are no additional clusters generated when the Zip4 is used, while Zip and SS aggregation generates one and two additional clusters respectively. There are no clusters in the real dataset that do not overlap with the clusters in the Zip4 dataset, while there are two, four, and four clusters that do not overlap with SS, CBG, and Zip clusters respectively. The similarity scores for the GeoMEDD clusters also display a similar trend as SaTScan clusters with Zip4 having the highest similarity (0.87) followed by SS (0.82), CBG (0.45), and Zip (0.17) clusters. The Zip4 and SS aggregation generates two additional clusters while CBG and Zip generates 5 and 32 additional clusters respectively. Similar to the SaTScan results, there are no clusters in the real dataset that overlap with the Zip4 clusters while there are one, six, and eight clusters from the real dataset that don’t overlap with the SS, CBG, and Zip clusters respectively.

**Table 8:** Similarity analysis results for SaTScan and GeoMEDD clustering.

	Category	Jaccard Similarity Score	Additional Clusters	Missing Clusters
SaTScan	Zip4	0.89	0	0
	SS	0.29	2	2
	CBG	0.01	0	4
	Zip	0	1	4
GeoMEDD	Zip4	0.87	2	0
	SS	0.82	2	1
	CBG	0.45	5	6
	Zip	0.17	32	8

### Privacy

#### Re- engineering risk with Zip4 aggregation

The distance-based re-engineering assessment on Cuyahoga County dataset indicate that about 20% of the spatial point data is assigned to a Zip4 centroid which is at the same location as the original (though these would still be a different mailing residence due to the congregate nature of the building). For the CBG, Zip, and SS categories, these values are 0.01%, 0.0003%, and 3% respectively. For Portage County, the corresponding values are 15%, 0.001%, 0%, and 2.9% for Zip4, CBG, Zip, and SS categories respectively.

#### Spatial K-Anonymity

**Fig 6:**
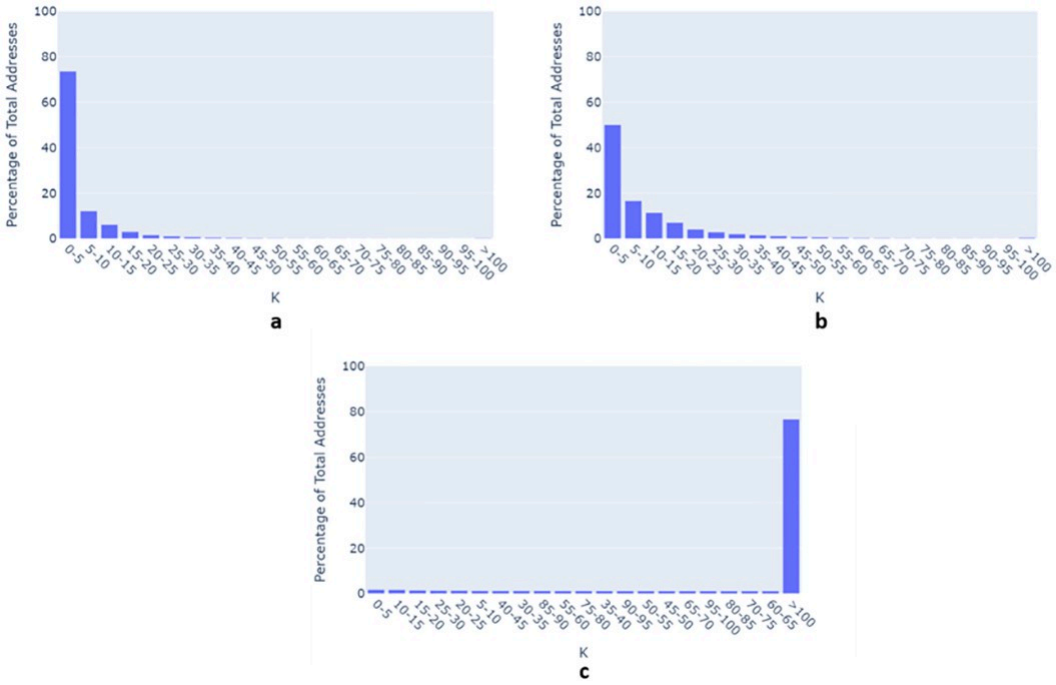
K value distribution with respect to the percentage of total addresses for (a) Zip4, (b) Street Segment, and (c) Census Block Group.

For the Zip4 based anonymization, the average k value was 6.3, meaning there was on average just over 6 other locations that could be chosen along with the original location. For a comparative analysis, k-anonymization was also implemented on the SS and CBG datasets. The average k-value for anonymization based on the SS and CBG centroid was 10.5 and 383 respectively. A histogram plot of K vs percentage of total addresses (Fig 6) for the three datasets show that the Zip4 dataset has a large number of addresses with a k-value within five (73%) (Fig 6a) followed by SS (Fig 6b) (50%). CBG has the highest number of alternative locations with a k-value being above 100 (Fig 6c) (76%).

## Discussion

As hospitals, public health departments and other health focused groups wish to join together to address emerging issues, especially when there is a need for granular sub-neighborhood scale operational support, so it becomes important to be able to share data safely while preserving analytical potential. In this paper, we have described one aggregation, the Zip4 that is already being used, even if somewhat unknowingly, as a means to share health data as a geographic layer. The analytical potential of this aggregation and the associated confidentiality concern is largely unknown in both the professional and research world. In this paper, we have shown that this aggregation could be more frequently and *knowingly employed* if the threshold of use is only set at not revealing the exact address of where a patient resides. At the same time enough spatial precision is preserved to allow for meaningful spatial analysis, even granular clustering that can support near-real time health intervention. We have also shown that spatial aggregations to coarser spatial units such as postal code (Zip), or Census Block group centroid do indeed provide better anonymization than the Zip4, though at the expense of analytical insight. What these results reaffirm is that continuum between aggregation, confidentiality and operational usefulness. What is different is the finer aggregation potential and risk of the Zip4 is not normally part of that continuum. Indeed, for many working in the field of health data analytics, the Zip4 would not even be thought of as an aggregation.

This new risk in data use is also not geographically homogenous, as was shown with the analysis of the line segments (Table 3) revealing a rural to urban differentiation. Cuyahoga County, which is predominantly urban, has relatively short Zip4 segments (91% within 200 meters), when compared to Portage County (67% within 200 meters) which is predominantly rural. The spatial distribution of the Zip4 segment lengths (Fig 1) also show a clear rural-urban pattern within the county, with most of Cuyahoga County having shorter Zip4 segments but these lengthening to the east and the more rural border with Geauga County (a predominantly rural county). On the contrary, Portage County, barring areas around its smallish towns, has relatively long Zip4 segments. This finding is similar to the published literature about the variations of postal code sizes between urban (small area with large population) and rural areas (large area with less population) [67].

Similarly, the distance distributions between the original location and the corresponding Zip4 centroid (Table 4) also display a similar pattern to the length distribution with Cuyahoga County having shorter centroid to actual location distance when compared to Portage County. Therefore, the spatial variation in the length of Zip4 segments indicates that the loss in accuracy is highly dependent on the underlying geography [17]. It should also be remembered, however, that in more dense areas, this geographic distance is complicated by a vertical distribution, the most obvious example of which are the multiple floors on a tower block. In other words, the final output map, even a weighted map would change little, though actually revealing an actual residence would still be hard to achieve.

The comparative spatial analysis results (Tables 5–7) clearly indicate that Zip4 spatial aggregation tends to preserve the underlying spatial structure when compared to other commonly used spatial aggregation units. This is vital when the need is for an analysis that loses little spatial precision, such as is the case with GeoMEDD [4]. One of the key results from the spatial analysis experiments is that the Zip4 spatial aggregation preserves both the same geometry (Figs 3 and 4) and content (members) (Table 8) of its spatial clusters. We attribute this to the property of the Zip4 preserving the underlying spatial structure. The higher similarity scores between the clusters generated from the Zip4 aggregated data and the real clusters clearly indicate that the cluster output is “correct” even after the spatial aggregation. Likewise, no real clusters are missed (Table 8). In a real-world scenario, a missing cluster could result in a potential delay in intervention. A further problem associated with spatial aggregation and spatial clustering is the generation of spurious clusters. The artificial congregation of points on the same location (which happens when centroids are used) generates clusters, which could potentially become a false alarm during spatial surveillance. Our results (Table 8) indicate that Zip4 spatial aggregation tend to generate a lower number of artificial clusters when compared to other spatial aggregation units.

However, while Zip4 spatial aggregation preserves spatial accuracy, our privacy experiments revealed that there are concerns regarding the potential for reengineering location. The distance analysis between the real and aggregated locations shows that approximately 20% of the addresses remain at the same location after Zip4 aggregation. While this finding appears to threaten the spatial privacy and confidentiality of patients it should again be stressed that these are apartment complexes, care homes, and nursing facilities with the same Zip4 code, so a different form of vertical obfuscation is at work. Indeed, while the spatial k-anonymity analysis (Fig 6) suggests reengineering risk, with an average k-value of 6.3 and 73% of address having a k-value below five, these calculations do not take into account the density at a single location [68]. While we would not recommend the sharing of maps displaying these Zip4 results, for collaborators working together within a secure space, where often the single most important concern for the organization or an Internal Review Board is to not include an exact identifier, this level of aggregation meets an acceptable threshold.

Even though we have performed an extensive set of spatial experiments, there are some limitations associated with our study. Firstly, we ran our experiments on simulated data and the clusters generated were only one of many possible scenarios associated with an outbreak. Secondly, we have only compared the Zip4 spatial aggregation to other centroid based spatial aggregation strategies, while there are many other advanced geomasking strategies such as the donut method, Linear Programming (PG) that obfuscate points both deterministically and stochastically. Thirdly, Zip4 codes can change and some of the Zip4 codes that we have used for this study might have changed as of today. To address these and other limitations in future work we plan to compare Zip4 to other advanced geomasking strategies so that standardized rules of the risk and benefits of different aggregation methods can be widely developed for health institutions.

## Conclusion

The fragmented health landscape in the United States, whether that be competing hospital systems or different jurisdictional health departments, need an effective means of spatial data sharing that can be used to pool resources to address an emerging crisis. Likewise, being able to share data with outside expertise, for example with academic spatial or data scientists, or even being able to comprehend the broader geographic situation of any health condition through a health clearing house, or also common needs. The long-standing challenge has always been the trade-off between spatial analytical (and operational) on-the-ground detail and not violating levels of acceptable confidentiality. While this problem has been frequently discussed, a largely ignored spatial aggregation, the Zip4 centroid, offers an interesting alternative in terms of greater analytical insights, but at the expense of having to rethink how we frame privacy expectations.

Adding the Zip4 to a sharable dataset happens frequently as many believe this is just a geographic segment designed to aid postal delivery, a misconception (in terms of analytical potential) due to a its general lack of utility in more commonplace spatial software, such as a GIS. In this paper we have shown that this aggregation does in fact produce a level of spatial detail that improves on the more typical spatial aggregation used to share data. The distance-based analysis described in the paper revealed a geographically varying level of positional accuracy when comparing the Zip4 centroids to the real address. This variation was tied to the underlying population density, with precision being much higher in more typical urban areas, and tapering off to less dense rural areas. Even so, the underlying denominator populations, or rather the number of residential addresses is likely to be more similar, and therefore offer similar masking capabilities, the mapped footprint looks far more problematic often being a single building. Therefore, especially in urban areas, the Zip4 aggregation preserves the spatial structure of the data as compared to street segments, Census block groups or Zip5 codes. Likewise, Zip4 centroids are also better at mirroring the positional accuracy of clusters and their memberships (a similarity of 89%). Additionally, this aggregation tends not to lead to the generation of spurious clusters or the suppression of real clusters, which is a highly desirable property for operational research. Simply put, this level of spatial detail not only helps better understand emerging patterns, but also situates the cluster in its more natural setting which can help explain causality and improve the logistics of intervention. However, this improvement comes at the cost of the way we more traditionally consider spatial confidentiality. The results of the spatial privacy experiment revealed that a low average value for k-anonymity (6.3%) and a higher percentage of (73%) aggregated addresses with a k-value below five indicates that re-engineering “risks” are relatively higher with Zip4 based aggregation. But, as previously mentioned, for more dense urban environments where this risk is greatest, a different form of masking occurs through the increased volume of residences within a single space. This leads to an interesting future debate regarding what is deemed as acceptable, especially between two different data sharing partners.

This paper has begun this debate with regards the potential, and risk, of sharing Zip4 data. These data are already often being shared due to a level of geographic data ignorance, at least in part due to more traditional spatial software, such as a GIS, still not being able to analyze or map this aggregation in a meaningful way. Though this is changing. What we have shown here is that the narrative around this change should be embraced as the potential advance it offers is great, as long as we clearly outline what the associated confidentiality risks are, including whether we should reframe how we define those risks. Data sharing in this form may prove to be one of the most significant advances in the spatial data analytics landscape.
